# Inverse Feature Learning: Feature Learning Based on Representation Learning of Error

**DOI:** 10.1109/access.2020.3009902

**Published:** 2020-07-17

**Authors:** BEHZAD GHAZANFARI, FATEMEH AFGHAH, MOHAMMADTAGHI HAJIAGHAYI

**Affiliations:** 1 School of Informatics, Computing, and Cyber Systems, Northern Arizona University, Flagstaff, AZ 86001, USA; 2 Department of Computer Science, University of Maryland, College Park, MD 20742, USA

**Keywords:** Representation learning of error, inverse feature learning, classification

## Abstract

This paper proposes *inverse feature learning* (IFL) as a novel supervised feature learning technique that learns a set of high-level features for classification based on an *error representation* approach. The key contribution of this method is to learn the representation of error as high-level features, while current representation learning methods interpret error by loss functions which are obtained as a function of differences between the true labels and the predicted ones. One advantage of this error representation is that the learned features for each class can be obtained independently of learned features for other classes; therefore, IFL can learn simultaneously meaning that it can learn new classes’ features without retraining. Error representation learning can also help with generalization and reduce the chance of over-fitting by adding a set of impactful features to the original data set which capture the relationships between each instance and different classes through an error generation and analysis process. This method can be particularly effective in data sets, where the instances of each class have diverse feature representations or the ones with imbalanced classes. The experimental results show that the proposed IFL results in better performance compared to the state-of-the-art classification techniques for several popular data sets. We hope this paper can open a new path to utilize the proposed perspective of error representation learning in different feature learning domains.

## I. INTRODUCTION

Recent feature learning trend and its branches such as deep learning have offered remarkable performance in image, speech, and natural language processing. Supervised or unsupervised representation learning are generally based on elements such as restricted Boltzmann machines (RBMs) [1], autoencoder [2], [3], convolutional neural networks (ConvNets) [4], sparse coding [5], [6], and clustering methods [7]–[9]. In the majority of existing supervised learning and representation learning methods, the term error refers to a function of the differences between the true and the predicted labels (i.e., loss functions). The error is utilized to optimize the training process or the learned features (e.g., optimizing the weights of neural nets). However, the notion of error can be considered in a more general term as a dynamic quantity that can capture the relationships between the instances and the predicted labels.

In this paper, we introduce the concept of error representation learning and propose a new framework for error generation and analysis called *inverse feature learning* (IFL). IFL is a feature learning method based on error representation to learn an additional set of impactful high-level features. The error representation learning is inspired by human’s decision-making process that involves analysis and inference of the results of his decisions. We believe proper error analysis to interpret error in the form of high-level knowledge is one of the missing puzzle pieces in the literature of feature learning. Such a perspective is somehow inspired by inverse reinforcement learning [10], which attempts to learn the reward function instead of the optimal policies. IFL interprets error representation in the form of several variables which depend on the predicted labels and the instances rather than the traditional notion of scalar values (e.g. loss functions). In other words, IFL is a supervised feature learning approach that learns a new set of high-level features by analyzing the interactions of instances and classes in a trial approach. The trial approach investigate the impact of assigning the instances to different labels.

To the best of our knowledge, IFL is the first work that performs feature leaning based on the perception of error representation. IFL proposes a new framework of learning by analyzing the interactions of instances and classes in a trial approach. During this trial phase, all possible labels are assigned to a test instance in order to generate the required perspective between the instances and each label in the form of new features. The key motivation of this paper is to introduce a new perspective for error representation in representation learning, and this basic proposed model can be improved in several levels and be applied in other domains.

## II. RELATED WORKS

Representation learning refers to a set of techniques which involve learning features from raw data to improve supervised, unsupervised, or semi-supervised learning approaches [11], [12]. Representation learning techniques have been utilized in several domains including speech recognition, signal processing, natural language processing, and text classification [11], [13].

The current methods in supervised or unsupervised representation learning are developed based on the representation of data. Some of the popular representation learning methods include ConvNets, RBMs, autoencoder, clustering methods, and sparse coding [1]–[9]. Supervised representation learning typically refers to a class of feature learning methods where the features are learned using the labeled data in a closed-loop manner. Deep learning techniques, as a subcategory of representation learning, learn a set of compact and high-level features of each class through multiple layers.

In recent representation learning methods, the unsupervised feature learning is usually used for pre-training nets, generally for deep networks, or for extracting high-level features, denoising, and dimensionality reductions. The authors in [14] used unsupervised learning for pre-training deep supervised networks, deep belief networks. Convolutional deep belief networks have been used for audio data in [15] and image processing in [7]. The authors in [8] used K-means as a clustering approach for each layer of the neural networks sequentially in a bottom-up approach and in an end-to-end way in [16]. Besides the aforementioned applications of unsupervised learning methods, clustering is also used or combined with classification methods. An example of combining clustering with classification is in the form of ensemble learning [17], [18]. Clustering methods can enhance the performance of classification techniques (even for shallow networks) through feature construction or extraction approaches [19]–[21]. For instance, the centers of clusters can be used as meta-data points or representative of instances inside a cluster [8], [22]. The authors in [23] developed a representation learning method for quasi-periodic signals based on clustering, where a set of high-level features were learned from the obtained clusters and used for classification.

Here, we would like to point out the distinctions of the proposed IFL techniques related to some common trends in machine learning. The proposed error representation learning method learns high-level features depending on the instances and classes. This IFL method generates error by trial and calculates the resultant representation of the error. The proposed method is considerably different from existing techniques in the literature since they focus on data representation learning and calculate errors by the loss functions that take the difference between the predicted labels and true labels.

This proposed method is different from generative adversarial nets (GANs) [24], which use two separate neural networks competing against each other with the ultimate goal of generating synthetic data similar to the original data inputs through the generator. This proposed method is also different from similarity learning [25], which learns a similarity function to measure how similar two objects are, in the sense that it extracts the relationships between the objects and the classes. Self-supervised learning methods are based on finding patterns inside of input instances [26].

The semi-supervised learning and active learning methods that utilize a combination of classification and clustering are based on the assumption that the instances which are in the same cluster have the same label and using this assumption toward predicting the labels for new instances [27]–[30]. In these methods, the instances near the center of clusters are considered as the most representative objects to determine the labels. Other approaches including [31], [32] utilized clustering for active learning in several different ways. The proposed IFL technique utilizes clustering for error representation learning in an innovative framework. Error representation learning is based on error generation and analysis scheme in which the instances are included in a cluster-based representation of different classes in order to observe the relationships between the inserted instances and different members of each class and the caused effects of that insertion.

Metric learning, similarity learning, techniques in semi-supervised learning, and adversarial autoencoders like GANs learning are based on data representation learning with the same traditional notion of error that refers to calculating the differences between the true labels and the predicted ones. However, our proposed method learns the representation of error that is intentionally generated in a trial process to generate an error for each class and to process this novel notion of class-dependent error in the form of features. The aforementioned methods do not generate and process representation of error for each and all classes simultaneously as a reference but rather use the error as a by-product of true and predicted labels. Therefore, our method develops a novel concept for error representation.

## III. INVERSE FEATURE LEARNING

**FIGURE 1.**
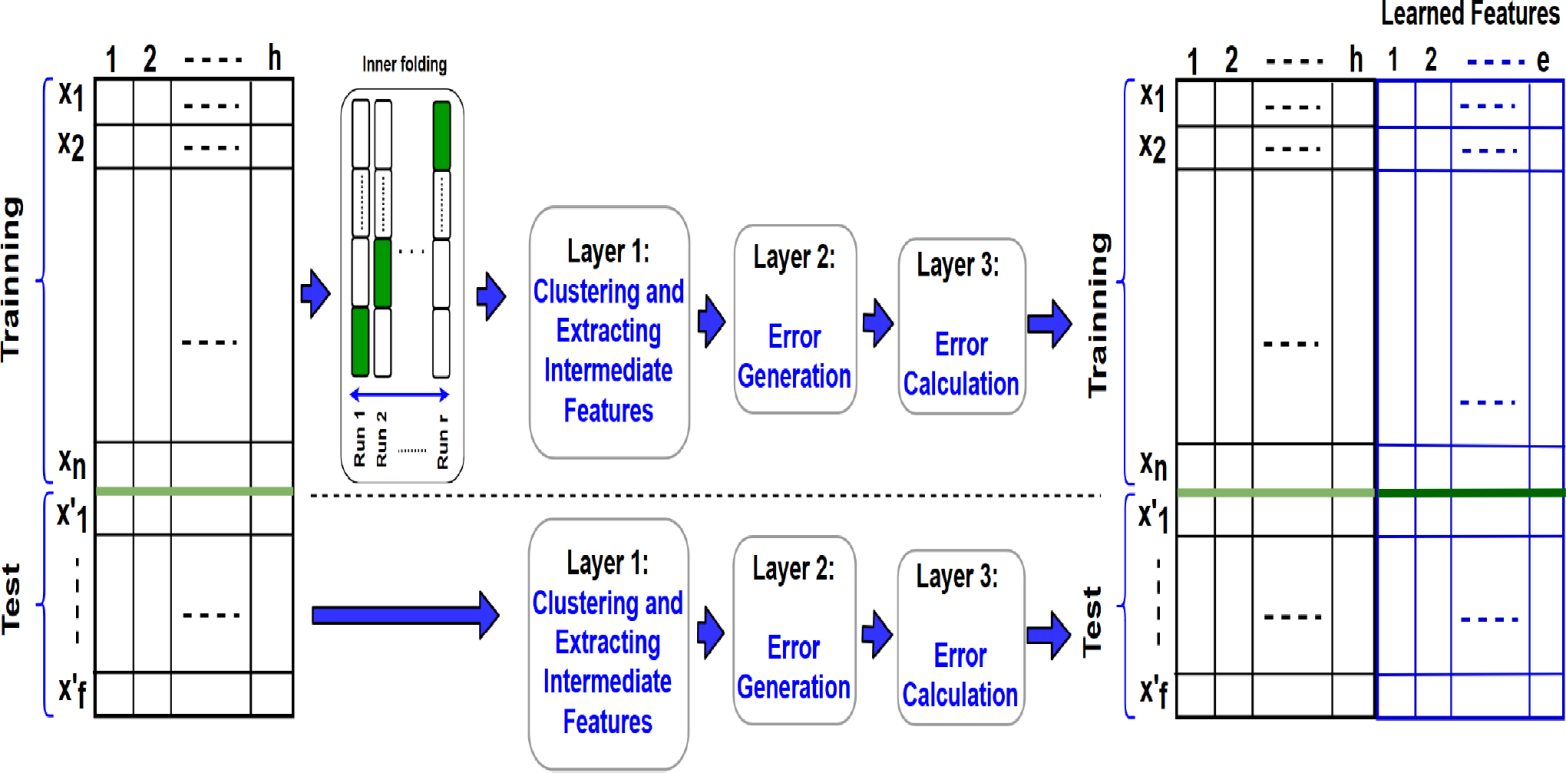
Block diagram of the proposed IFL method. The figure demonstrates the feature learning process for both training and test sets. The upper part of the figure depicts the inner-folding for the training set during *r* rounds. The one fold of the inner-test in each run is highlighted by green. The lower part of the figure demonstrates the feature learning process for the test data set.

In this section, we introduce the proposed IFL mechanism that learns the representation of error using a trial approach. The operation of this method for training and test instances is described in the following sub-sections III-A and III-B, respectively. The overall block-diagram of this method is shown in figure 1.

### A. ERROR REPRESENTATION LEARNING FOR TRAINING INSTANCES

The objective of the IFL method is to learn a set of additional features per sample by trial to extract the relations between the sample and the classes. This process is performed during two phases for the training and test sets. In each phase, the samples are assigned to the *set of samples of the available classes* one at a time and the changes in the characteristics of data before and after adding each sample are analyzed. Here, we provide an overview of the proposed method.

First, in inner folding phase, we partition the training instances to inner-training and inner-test sets during each fold in such a way that each training instance is considered as an inner-test instance once. Then, in layer 1 of the proposed IFL, the inner-training samples with the same labels are grouped together. Next, the groups of samples (i.e., the samples with the same label) are clustered to a pre-determined number of clusters. The representation of these clusters for each label are calculated in the form of several intermediate features. In layer 2, each inner-test sample is intentionally assigned to all available classes, and then one of two described strategies are performed to extract and analyze a notation of error as a means to learn a new set of high-level features. In layer 3, regardless of the fact that the sample has been assigned to the right or wrong classes, we measure two sets of metrics per sample for each class. These two sets of learned metrics are then added to the original data as the learned features. Since these features are learned per class, the features belonging to different classes need to be separated from one another. Therefore, we introduce two techniques that depending on the number of instances and the number of classes are used. The model is trained and evaluated by adding the learned features per instance to the set of the primary features of the corresponding instance for both training and test instances, respectively. In the feature learning for training instances, the features are only learned for inner-test samples, in which their labels are not considered in the process. The reason is that we aim to develop a unified framework for feature learning during the training and test phases in classification, where the test instances do not have labels.

**FIGURE 2.**
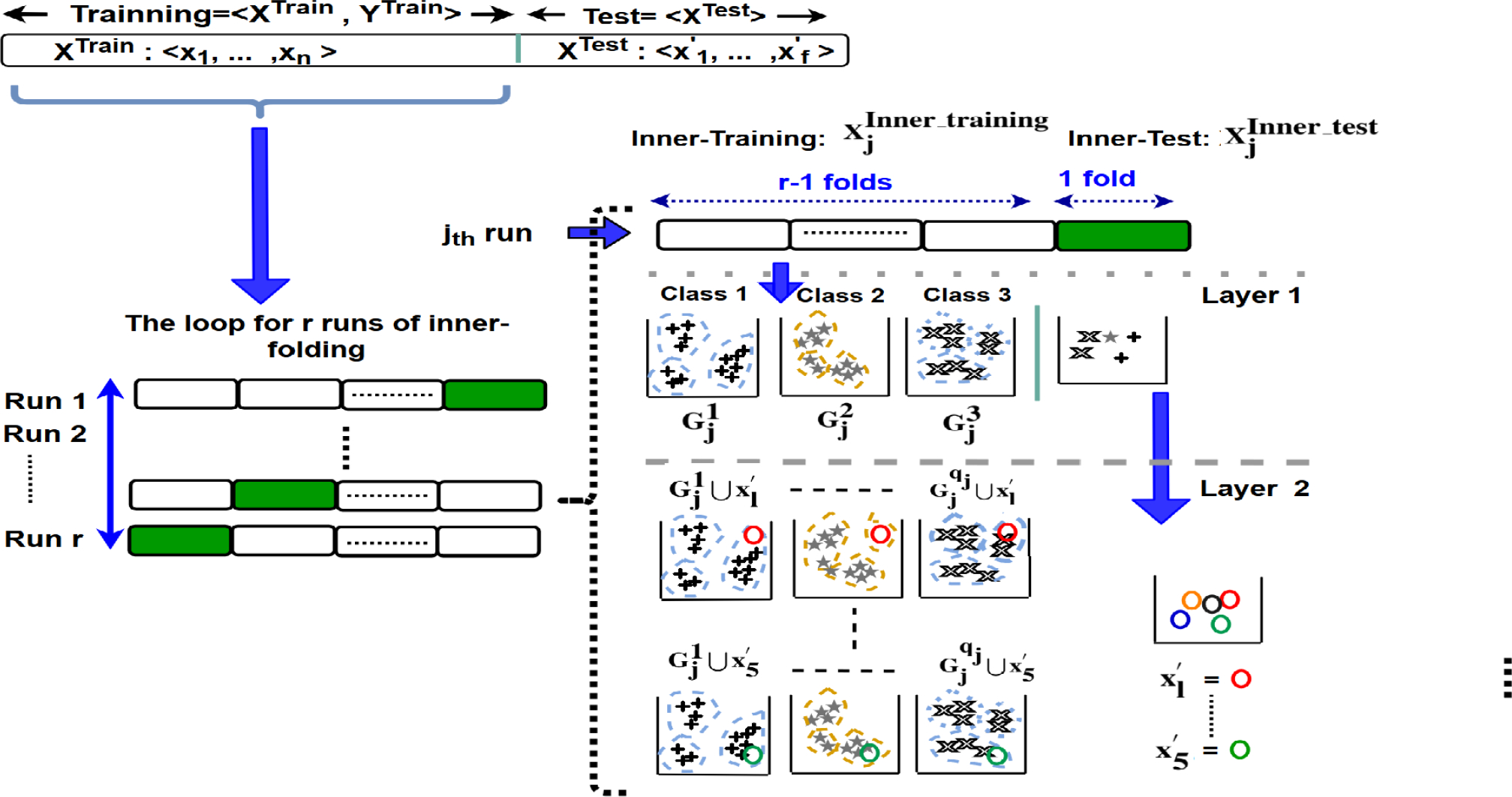
Diagram of the main blocks of the proposed feature learning process for the training data set including the inner-folding, layer 1 (clustering) and layer 2 (adding the inner-test samples). In this example, the number of classes in round *j* is assumed as 3 (i.e., *q*_*j*_ = 3). It is also assumed that the one fold inner-test includes five samples. These samples are denoted with circles in layer 2.

To formulate the problem, the input training data set is presented with *D* 〈*X*^*Train*^, *Y*^*Train*^〉, which *X*^*Train*^ = {*x*_1_, ⋯ , *x*_*n*_} indicates the set of input training instances and *n* shows the number of input instances in the training partition. Each instance *x*_*i*_ = 〈*x*_*i*,1_, ⋯ , *x*_*i*_,_*h*_〉 consists of *h* features. The label set is denoted by *Y*^*Train*^, where *Y*^*Train*^ = {*y*_1_, ⋯ , *y*_*n*_} is a vector corresponded with data set *X*^*Train*^. Thus, *y*_*i*_ shows the corresponding label for *x*_*i*_. Since we focus on classification, the labels are categorical. $Z=\langle z_{1},\;\cdots \;,z_{m}\rangle$ shows the set of classes, in which *m* indicates the number of classes. $X^{\text{Test}}=\langle x_{1}^{\prime },\;\cdots \;,x_{f}^{\prime }\rangle$ denotes the test set in which *f* refers to the number of test instances. Notation |*b*| indicates the number of instances in set *b*. In continue, we describe the building blocks of this method with more details, as depicted in figure 2.

#### 1) INNER FOLDING

The first step is to find the representation of samples belonging to different classes for both training and test sets. It is simple to obtain the representation of each class for the test samples as we can simply partition the training samples to different classes in order to obtain the representation of each class. However, the equivalent process of partitioning is more computationally complex for training samples, since each sample of the training set should be considered against all remaining samples with a strategy similar to leave-one-out cross-validation. In each case, the number of remaining samples to learn the class representation is *n* – 1, in which *n* denotes the number of training instances. Hence, this process involves a large number of repetitions of the feature learning process (i.e., *n* times) that is not scalable to large data sets. Therefore, we instead apply a folding mechanism, here called *inner folding* to only perform the feature learning process for a limited number of runs (i.e., *r*-runs, where *r* ≪ *n*). During each round of inner folding, the training samples are divided into two partitions of inner-training and inner-test samples.

We introduce inner folding as a partitioning mechanism that works similar to *k*-fold cross-validation in terms of partitioning data, but the objective of this inner folding is different from typical cross-validation folding methods. Inner folding is a framework that partitions 〈*X*^train^, *Y*^train^〉 to *r* folds. It runs *r* times, wherein each run, *r* – 1 folds are used for training and 1 fold is used for test. Here, each fold is considered as a test fold only one time. The training and test partitions in each run are called as *inner-training* and *inner-test*, respectively. Thus, inner folding is different from cross-validation since it is used as a mechanism to evaluate the characteristics of one test sample of inner-test against the inner-training samples in each run.

The inner-training and inner-test sets of the *j*^th^-run of the inner folding are shown with 〈 $X_{j}^{\text{Inner\_train}}$, $Y_{j}^{\text{Inner\_train}}$ 〉, and $\langle X_{j}^{\text{Inner\_test}}\rangle$, respectively. Clearly, $X^{\text{Train}}=X_{j}^{\text{Inner\_test}}\cup X_{j}^{\text{Inner\_train}}$. The trial process involves assigning each test instance, $\forall x_{f}^{\prime }\in X_{j}^{\text{Inner\_test}}$, to each available label, *z*_*i*_, $z_{i}\in Z$ that exists in $Y_{j}^{\text{Inner\_train}}$. In continue, we describe the three layers of error representation for each run of the inner-folding process.

#### 2) LAYER 1: CLUSTERING AND EXTRACTING INTERMEDIATE FEATURES

As mentioned before, the proposed IFL method is designed based on the analysis of error representation. The error is measured using different metrics after the assignment of the inner-test samples to different classes in order to investigate the variations in the relative relations among the samples. To do that, first, the instances of each class are clustered to a pre-determined number of clusters using *K*-means algorithm as an unsupervised learning method. K-means algorithm is selected as the clustering technique since it is very fast and can be scaled to high-dimensional data sets [33]. However, we understand that *K*-means algorithm does not perform well in cases, where the instances have different densities or the instances are distributed in non-spherical forms. We should note that the IFL method is generic and can be implemented with other clustering techniques.

During each round *j* of the inner-folding process, the instances of $X_{j}^{\text{Inner\_train}}$ are first categorized based on their labels, $Y_{j}^{\text{Inner\_train}}$, to $G_{j}=\langle G_{j}^{1},\;\cdots \;,G_{j}^{q_{j}}\rangle$, in which *q*_*j*_ shows the number of labels in $Y_{j}^{\text{Inner\_train}}$. The corresponding input instances and labels of group *i* during round *j*, $G_{j}^{i}$, are shown with $X_{j}^{\text{Inner\_train,}i}$. Then, the samples of each class *i*, $G_{j}^{i}$, are clustered to *k* clusters. These clusters are ordered based on the number of their member instances and shown with $C_{G_{j}^{i}}=\langle C_{G_{j}^{i}}^{1},\;\cdots \;,C_{G_{j}^{i}}^{k}\rangle$.

During the clustering, each object is assigned to the cluster that has the nearest centroid as a mean of its instances based on a distance metric. The objective function of K-means is considered to find the centroid_1_, … , centroid_*k*_ in order to minimize the objective function, *O*, as described in (1).

(1) $$O=\overset{k}{\underset{t=1}{\sum }}\overset{s}{\underset{l=1}{\sum }}\|x_{l}^{t}-{\text{Centroid}}_{t}{\|}^{2}$$

where *s* denotes the number of instances in group $G_{j}^{i}$ with label *i*, and $X_{l}^{t}$ denotes the instance *x*_*l*_ that belongs to cluster *t*.

*Mean-group* is a metric defined as the mean of each group, $G_{j}^{i}$. This metric, as described in algorithm 1, is a vector denoted by $\mu_{j}^{i}$ that its *l*^th^-element is calculated as the average of *l*^th^ features of all instances that belong to this group.

In order to evaluate the characteristics of the clusters of each class, we define three other metrics of *confidence*, *mean*, and *centroid*, as described in algorithm 1. These measures act similar to kernel functions of ConvNets to extract the representation of each cluster. The *centroid* indicates the center of a cluster that is obtained by the clustering method. The *Confidence* is a singular scalar value and defined as the ratio of the number of instances of each cluster to the number of all instances of that class. In other words, the confidence metric is a membership value that shows the probability that an instance belongs to a particular class. The *mean* is a vector with length *h* that calculates the average of *l*^th^ features of all instances that belong to a cluster. The calculation of these measures in layer 1 captures the baselines to learn the error as defined in layer 2.

#### 3) LAYER 2: ERROR GENERATION

In machine learning methods, error is the result of differences between the predicted output and true output that is measured by loss functions and used to train the model. In a simple case, the error is “one” when a predicted label is incorrect, and the error is “zero” when the predicted label is correct. In the proposed IFL method, instead of using this traditional notion of error, the error is measured based on a resultant representation of assigning the instances to different classes in a trial approach.

As mentioned earlier, the instances that belong to each group $G_{j}^{i}$ of $G^{j}=\langle G_{j}^{1},\;\cdots \;,G_{j}^{q_{j}}\rangle$ have the same label. In layer 1, the representation of each class and its clusters were measured using several intermediate features (e.g. the mean of a class, and the centroid, the mean, and the confidence of the clusters). The goal of layer 2 is to evaluate the changes in these intermediate features, and in a more general sense, the representation of each class using the formed clusters by adding the test samples of inner-test. In other words, we assign the samples of the inner-test set to the existing labels one at a time. Therefore, the term trial refers to the process of inserting a new inner-test sample, $x_{l}^{\prime }\in X_{j}^{\text{Inner\_test}}$, to a group of samples with the same label, $G_{j}^{i}$ and generating a new set of $\left(X_{j}^{\text{Inner\_training},i}\cup x_{l}^{\prime }\right)$.


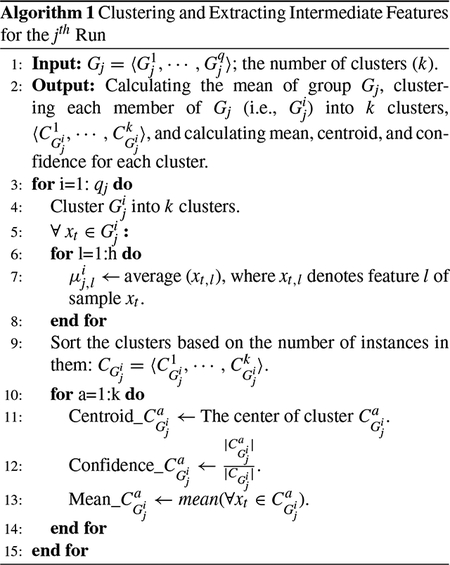


We have considered two strategies to evaluate the characteristics of data after addition of new inner-test samples. In the first strategy, the similarity between the instance and the centers of clusters of each class is measured to find the closest one in order to assign that instance to this closest cluster. In other words, this sample is added to the closest cluster. Thus, in the first strategy, which is the simpler option, is to calculate the distances between the sample of inner-test, $x_{p}^{\prime }$, $\left(x_{p}^{\prime }\in X^{{\text{Inner}}_{{\text{test}}_{j}}}\right)$ and the centers of all clusters in $C_{G_{j}}=\langle C_{G_{j}^{i}}^{1},\;\cdots \;,C_{G_{j}^{i}}^{k}\rangle$ (obtained in algorithm 1), and then assign $x_{p}^{\prime }$ to the closest cluster of the class *i*. This cluster is denoted by $C_{G_{j}^{i}}^{*}$. After that, the confidence, centroid, and mean are only calculated for the nearest cluster. This strategy is described in Algorithm 2.

In the second strategy, the formed clusters of layer 1, $C_{G_{j}^{i}}$, are no longer used. Instead, in this strategy, the set of instances in each class including the primary and new instances are clustered again. The first strategy involves fewer computations, however, it does not necessarily perform as well as the second strategy of re-clustering when the densities of clusters are considerably different. For instance, when there are several outliers in the original groups.


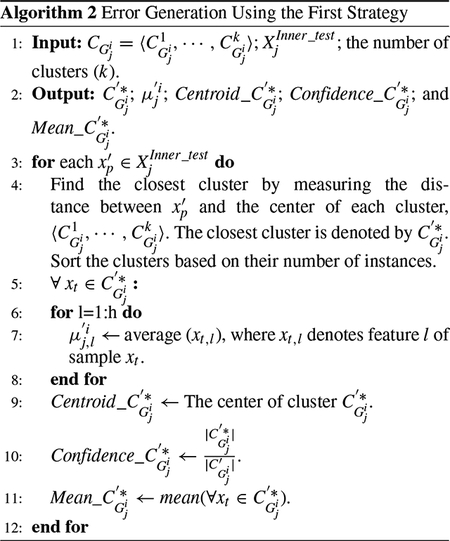


#### 4) LAYER 3: ERROR CALCULATION

The purpose of layer 1 was to capture the representation of classes in the form of clusters before the trial process. In layer 2, the representation of each inner-test instance and classes after the trial was captured when each inner-test instance is added to a cluster of each class. Now in layer 3, the representation of error is learned through several high-level features by non-linear modules as described in the following. These features capture the relationships between the inner-test samples and the clusters through two feature sets. The definition of these features are summarized in Algorithm 3.

##### Feature Set 1: The distances between the new instance and the clusters of each label

This feature set captures the representation of error inside of each class including the distance of this instance to its closest adjacent instance in the class (*feature*_1.0_), the distance of the instance with the mean-group (*feature*_1.1_) as well as the distance of the inner-test sample with the centers of all clusters for each class (feature set: *feature*_1.2_), the distance of the inner-test sample with the means of all clusters of each class (feature set: *feature*_1.3_).


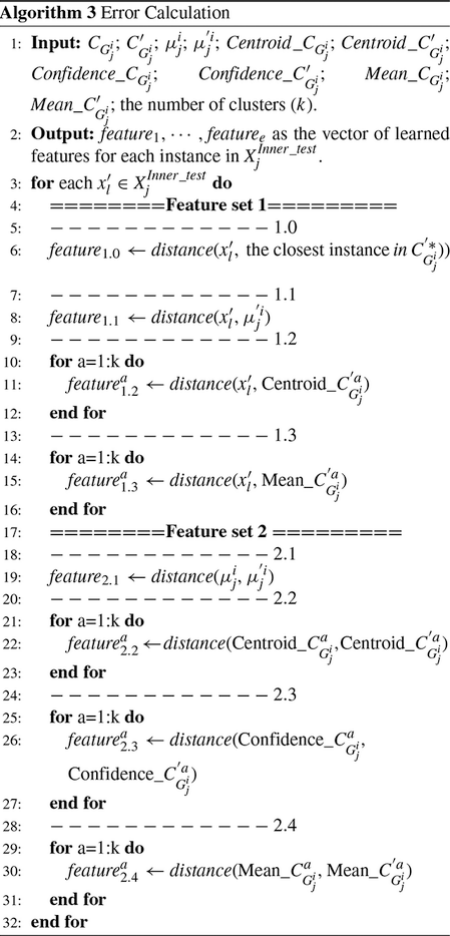


##### Feature Set 2: The changes in class representations before and after inserting the new inner-test sample

To capture the effects of error in the level of classes, the distances between the means and confidences of the clusters of each class, formed in layer 1 and layer 2, are calculated. Also, the distances between the centers and the means of clusters that were formed in layer 1 and layer 2 within each class are measured. We also measure the difference between the confidence metric of original cluster formed in layer 1 and the cluster including the sample in layer 2 that represents the membership value denoted by *feature*_2.3_.

Each instance in the training is considered as the inner-test instance for one time and then these features are calculated for this sample. The learned features in layer 3 are added to the set of the primary features of that inner-test instance. Thus, the output of the inner folding is a set of features per instance of the training. There are two techniques to join the learned features with the primary features. First, the learned features of each class are joined to each other and the primary features. Second, each instance is replicated as the number of classes and the learned features of each class are joined to one of the replications. If the number of instances and also the number of classes are few, we use the first technique. The second technique preferred if the number of classes or the number of instances are large enough. Since learned features of each instance are calculated for each class, they need to be differentiated from each other by a hint if the number of the instances is too few such as “Cryotherapy” data set in Table 1. Therefore, the features of each class are multiplied by the class ID to be separated from each other in which the number of instances is too few. For example, if we have three classes, a, b, and c, the learned features of these classes are multiplied to 1, 10, and 20, respectively to be distinguished from each other. The idea behind multiplying the learned features of different classes to a pre-determined number is to explicitly separate them from each other. In our selected strategy where the learned features of different classes are multiplied by a multiple of the classes’ ID, the set of corresponding features of each class can be distinguished by their values and this fact can be used as a key additional clue for the classifier. We use the multiplying just for “Cryotherapy” data set. We show the used technique for each data set in the experimental results. “No free lunch” theorem implies that there is no method that is superior to other methods over all data sets. We learn two sets of features that some features are selected depend on the data sets to have general abilities. We use ReliefF [41] as a feature selection approach for this purpose and we tune the number of features depending on each data set. Such separation of learned features of different classes cannot be easily handled by some classification methods, therefore, we utilized ensemble decision tree as the classifier for our approach to best treat this proposed embedded distance mechanism. In the following section, the process of feature learning for the test instances *X*^*Test*^ has been described.

### B. ERROR REPRESENTATION LEARNING FOR TEST INSTANCES

In this phase, as is shown in figure 1, the entire training set along with its additional learned features can be used as the training instances in the feature learning method (i.e., the inner folding process is no longer required). Therefore, the training and test instances are fed to the three-layer feature learning method to learn corresponding features for the test instances. We would like to note that the training instances are grouped based on their labels and then clustered. The test instances, which their labels are not available, are assigned to the proper cluster of each label and then the features are learned for each test instance. Hence, this step does not require the labels of test instances. Finally, the extended training and test data sets with the learned features are fed to the classifier.

### C. AN ILLUSTRATIVE EXAMPLE FOR IFL

In this section, we provide a simple illustrative example to explain the proposed IFL process. The idea of the IFL is inspired by the human decision-making process, where the expected outcome of every potential action is envisioned before making a decision. In the proposed IFL method, a trial process is designed in which through an inner-folding process, the instances for each class are clustered and then for each new inner-test instance, the possible outcomes of assigning different labels to this new instance is evaluated in the form of some learned features.

**FIGURE 3.**
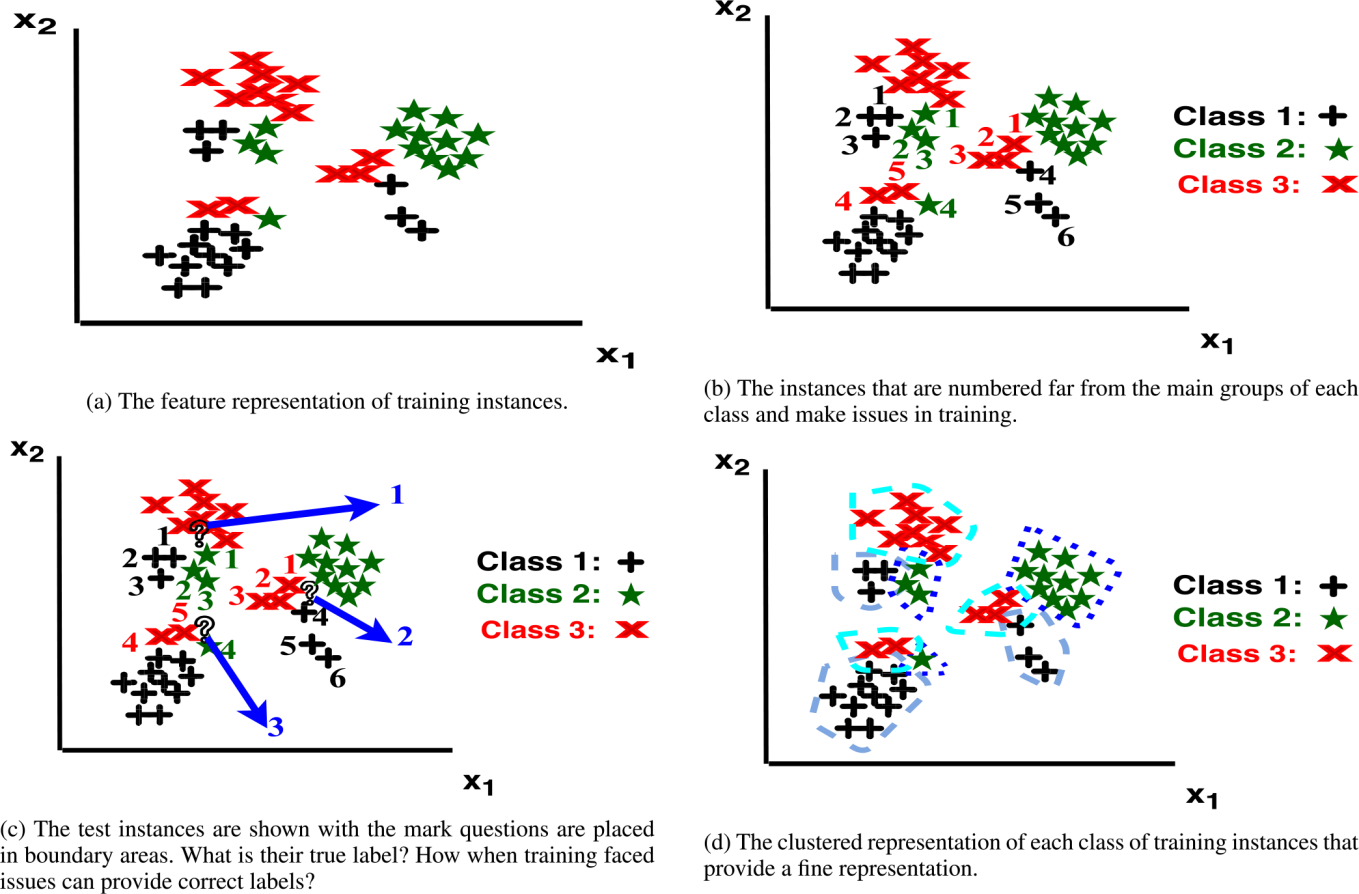
A challenging example for a classifier and how IFL capture the local and global representation.

Let us consider an example of a data set with two features, 〈*x*_1_, *x*_2_〉, and three classes as shown in figure 3a. In figure 3b, the instances from each class which are mixed with the samples from the majority of other classes are marked with numbers. In figure 3c, the instances that are shown with question marks are test instances that illustrate the case, where the test instances are added to the figure. It is a challenge to provide the decision function that minimizes the risk for a classifier to learn the labels of the instances that are marked with numbers as training instances in figure 3b. They are close to the groups of instances that the major of them belong to a different class and several instances of different classes that exist in that around. This example intends to demonstrate a scenario, where the test instances are likely to be misclassified by common classification methods.

We propose inverse feature learning that trial different assumptions of different classes one by one. First, we obtain a fine resolution of each class by a clustered representation as shown in figure 3d. Then, we follow a trial of different assumptions of belonging as shown in figure 2. The features that calculate characteristics of the instances to the centers of the closest clusters capture the local perceptive. The features that measure the characteristics of a selected cluster to all other clusters capture global perspectives. In other words, IFL features represent the possibility of each class considering the local and global aspects of the feature space. An instance is assumed to be assigned to each potential label for training and test instances. This method is specifically valuable to learn the representation of the samples which are close to the borders of other classes (see the numbered instances in figure 3b) during the training phase of the trial, as small clusters are formed for these samples to learn their relative representation related to the distributions of each class with a higher resolution. During the test phase, when the test instances (marked with a question mark) are given to the learning algorithm, their relations to the cluster representation of each class are calculated and used as additional information for classification. Now, IFL gives the importance to the numbered one of instances and the main portions of different classes based on what is learned through training.

## IV. TIME COMPLEXITY

The IFL method is developed based on clustering the data set using *K*-means algorithm through a trial process to learn several high-level features based on representation of error. For the sake of simplicity, this process is explained in a sequential way (algorithm 1), however, the process of clustering for different instances can be performed simultaneously. Therefore, the time complexity of this method would be a function of time complexity of the underlying *K*-means clustering algorithm.

The time complexity of Lloyd’s algorithm of *K*-means is *O*(*eknh*), in which *e* is the number of iteration, *k* is the number of clusters, *n* is the number of instances, and *h* is the number of features [34]. Since the number of iterations, *e*, the number of features, *h*, and the number of clusters, *k*, are constant, the time complexity of this algorithm is linear. In the second strategy of error generation, the samples in each class are clustered again after adding a new test sample as described in Section III-A3. Hence, the *K*-means clustering method is performed 2 × *r* times during the inner folding process, in which the number of runs, *r* ≪ *n*, is a constant number. Thus, the time complexity of the feature learning process is linear. Obviously, the time complexity of the first strategy in error generation, where the new instance is added to the cluster with the closest center is also linear.

## V. EXPERIMENTAL RESULTS

In this section, we evaluate the performance of IFL method using several popular binary and multi-class data sets. To do so, we compared its performance versus several classification methods that only use the original features. We embed the corresponding classes of learned feature sets of instances by two different techniques depending on the number of classes and the number of instances as described in III-A4. We use the first technique for “Cryotherapy”, “Diabetes”, “Heart”, and “Ionosphere” data sets in which the multiplication is applied just to the “Cryotherapy” data set because its number of instances is too few. We use the second technique for the other data sets. The classifiers that consider the whole features as a unified set are subject to a weak performance here. Thus, we used boosting decision trees as the main classifier for the proposed method because it works based on several decision trees that can better handle a bigger set of features of learned and original features corresponding to different classes compared to other classifiers. Different metrics including accuracy and F1 scores are used to evaluate the performance of our method. Also, we compared the performance of this IFL versus several deep representation learning approaches, as described in [35], such as Linear ELM [36], Deep Belief Networks [37], Stacked Auto-Encoder [14] for pre-training weights of the deep network alongside a softmax classifier [38], DrELM [35], and *DrELM*^*r*^ [35]. The results are reported using “Statistics and Machine Learning Toolbox” of MATLAB ®.

We also evaluate the performance of the IFL method in comparison with several feature selection, feature extraction, and feature transformation techniques which are applicable to the selected data sets. Feature selection methods remove redundant, irrelevant, and noisy features that help to make a balance between the number of features and number of instances for classic classification in small data sets. Feature extraction or learning methods which are based on learning new features from raw data still suffer from degraded performance to provide a useful inference in data sets with a small instance space and a large number of features [39], [40]. The feature extraction methods which are based on objective functions can construct and learn a new set of features to improve the performance. The performance of our proposed method is compared with several feature learning, feature extraction and feature selection techniques in Table 3. The same Ensemble classifier is used for these techniques as well as the IFL algorithm.

Feature selection (FS) methods are categorized into three groups: filter-based techniques that analyze the data independent of the classifier, the embedded methods which integrate the selection process into the learning the classifier, and the wrapper-based methods which measure the importance of the features based on the classifier performance. Among the filter-based FS methods, we have selected ReliefF [41] and Fisher [42]. ReliefF is a supervised approach that works in an iterative manner. Fisher as a supervised feature selection method calculates the importance of the features as the ratio of inter-class separation and intra-class variances [43]. In the Embedded category, we compare the performance of IFL with Lasso [44] and unsupervised discriminative feature selection (UDFS) [45]. In Lasso, the features are ranked by using K-nearest neighbors per class. The importance of features is measured based on their separation power in a linear SVM. UDFS as an unsupervised FS algorithm uses L2,1-norm regularized searches for the most discriminative feature subset in batch mode [46]. In the wrapper category, we select FSV [47] and dependence guided unsupervised feature selection (DGUFS) [48]. FSV works based on a linear programming technique through the training of an SVM. DGUFS selects the features and partition data in a joint manner that preserves the dependency among data, cluster labels, and selected features [48]. We select 90% of the most important of features as the input features of the Ensemble classifier and the remaining 10% features (redundant and correlated features) are discarded.

We also used two known feature extraction methods including sparse filtering (SF) algorithm [49] and reconstruction independent component analysis (RICA) Algorithm [50]. They learn transformations that map the features to new features. Sparse filtering algorithm and RICA attempt to minimize the sparse filtering objective function and Reconstruction ICA Objective Function by using a standard limited memory Broyden-Fletcher-Goldfarb-Shanno (LBFGS) quasi-Newton optimizer respectively. For example, the objective function of sparse filtering simultaneously learns several features for each instance as each learned feature has a similar weight [49], [50]. We evaluate the sparse filtering and RICA in two scenarios where the number of learned features added to the data set is equal to 20% or 50% of the number of original features.

We also compared the IFL method against principal component analysis (PCA) as a common feature transformation technique [51]. We consider two scenarios of using the PCA method with the threshold value of 99% and 99.9%.

We evaluated the results of our method over several runs for different data sets and the results showed very small variances. Theoretically speaking, since we just added a number of features and used the common classifiers that do not depend on stochastic behaviors; therefore, the results only have very small variances over different runs. The only stochastic part of the method is the clustering, where the clustering methods such as k-means can be easily stabilized [52], [53] and are used in feature learning methods [8], [9].

A number of common data sets with a different number of instances, features, and classes are used in this study including Cryotherapy, Heart, Segment, Magic, Letter, Credit, Spam, and Ionosphere [54]. The characteristics of these data sets are summarized in Table 1. The reported results are obtained using *k*-fold cross-validation. Let us first describe the parameters involved in the proposed method before presenting the results.

As described in Section III, the feature learning process for training data is based on a *r*-fold inner folding process, where *r* shows the number of folds. Each round of inner folding involves clustering the training data set using *K*-means, in which *k* is the number of clusters. We consider *k* equals with *r* in experiments and we use the City Block as the distance metric in clustering and feature calculation. The corresponding parameters used in the proposed IFL method for the results in Table 5 are described. The number of folds for cross-validation of the baseline classifier in Table 2 is selected as 5, 10, or 15 as used for the proposed method in the second column of Table 5.

The reported results are based on using the first strategy of error generation (i.e., assigning the test instances to the closest cluster). The results are reported for the case that the proposed IFL method is performed over boosting decision trees classifier. The reason behind selecting the boosting decision trees is its robust performance versus different feature sets per class since we embedded the label of each class in the learned features of that class. Different baseline classifiers such as Naive Bayes (NB), Decision Tree, *K*-nearest neighbors (KNNs), and an Ensemble Classifier in form of boosting decision trees, as the same classifier that we used for IFL, are used for the sake of comparison.

As it can be seen in Table 2, the proposed feature learning method provides considerably better results in different data sets compared to the known classifiers that only work with original features. It means that the learned features using our method can significantly improve the results of popular classifiers. The parameters of the proposed feature learning method are fine-tuned based on the data sets as described in Table 5. In Table 5, the fourth column describes the set of learned features (as described in Algorithm 3) that are used for each data set.

We also compared the result of the proposed method in terms of accuracy with several most known approaches that learn deep representation in Table 4. As can be seen in the table, the proposed method can provide comparable results with these methods or outperform for some data sets, while it is worth noting that our method can learn new classes in an incremental manner without modifying the learned features of previous classes. However, the deep representation learning generally cannot be easily scaled-up to learn new classes without re-training. Based on the well-known “no free lunch” theorem, there is no method that is superior to other methods over all data sets. As it can be seen in Table 4, for Diabetes data set, the accuracy of the IFL is just about 1% below than *DrELM*^*r*^ [35] and the accuracy for Segment data set is 2.2% better than the known deep representation leanings as the-state-of-art in recent literature.

As it is shown in Table 3, the performance of the IFL method is better than the feature selections and extraction methods. The results indicate that the IFL method is capable of extracting useful information from the data set that are not captured by common feature extraction, selection, and transformation methods. The comparison of the elapsed time of the classifier based on the primary features with the elapsed time of IFL calculation and the classifier for IFL is shown in Table. 6. The elapsed times are reported in seconds and the calculation of the features is done serially. Since the features are calculated for each instance depending on each class, it can be seen the differences become larger in which the number of instances is large. The calculation of the features in a parallel way depends on the available resource that can decrease the elapsed time considerably.

We should note that we evaluated the performance of this IFL method using other clustering techniques such as spectral clustering (e.g., DBSCAN [55]). Using this clustering method led to better results since it can handle different data densities in better forms. However, this clustering works based on a graph Laplacian matrix that requires a considerable memory to operate, therefore, it was not easily feasible for large scale data sets.

## VI. CONCLUSION

In this paper, we propose error representation learning as a new feature learning trend that deals with error as a dynamic component that can disclose a valuable set of information about the relations of the instances and the classes. To the best of our knowledge, current machine learning methods interpret the error in a simple notion of a constant scalar that evaluates the differences between the true and the predicted labels. In this paper, we propose a general concept of error representation that can evaluate the error in several new levels in order to learn high-level and explicable features by trial. The proposed feature learning method based on error representation, called *inverse feature learning* adds the set of learned features to the set of primary features. The IFL method is performed in a hierarchical structure by evaluating the results of adding each instance of interest to available classes using several different metrics. The experimental results show the significant performance of this feature learning method compared to several state-of-the-art classification methods and most known deep representation learning methods. Another clustering strategy to handle large scale data sets is using deep learning deep-learning-based clustering methods. However, the deep learning strategy cannot learn classes simultaneously, be applied on small data sets, and learn new classes without learning from scratch. We consider developing a deep-learning-based clustering approach for inverse future learning as our future work [56].

## Figures and Tables

**TABLE 1.** The specification of data sets.

Data sets	#Instances	#Features	#Class
Cryotherapy	90	7	2
Diabetes	768	8	2
Heart	270	13	2
Credit	30000	23	2
Segment	1500	19	7
Ionosphere	351	34	2
Glass	214	9	6
Spam	4601	57	2
Magic	19020	10	2

**TABLE 2.** The comparison of accuracy and F1 score of the baseline classifiers and the IFL. We have evaluated the performance of data sets with several popular classifiers libraries in MATLAB and sklearn including SVMs and logistic regressing but only the best results among different classifiers are reported. In the proposed method, the learned features are independent of the classifier, hence using a better classifier can achieve higher accuracy over our learned features as it does over other sets of features.

#	Data sets	Naive Bayes		KNN		Decision Tree		Ensemble		IFL	
		F1	Accuracy	F1	Accuracy	F1	Accuracy	F1	Accuracy	F1	Accuracy
1	Cryotherapy	91.09	90.98	89.94	90.54	92.21	93.21	86.56	87.91	**96.65**	**96.67**
2	Heart	79.10	78.64	79.75	81.15	76.27	78.27	77.73	78.90	**81.82**	**82.22**
3	Segment	88.98	88.44	96.07	96.00	93.93	94.39	97.62	97.61	**97.97**	**98.00**
4	Glass	53.96	61.55	74.77	75.12	65.71	69.25	70.85	74.70	**76.52**	**79.44**
5	Magic	69.07	76.03	80.13	82.44	80.84	82.14	85.49	86.99	**86.07**	**87.61**
6	Diabetes	67.97	71.93	65.69	69.50	68.25	71.18	68.83	71.81	**73.31**	**76.56**
7	Ionosphere	90.19	89.61	89.56	90.11	86.91	88.35	92.43	92.57	**94.99**	**95.44**
8	Spam	51.47	52.41	91.29	91.84	91.27	91.86	94.64	95.52	**95.44**	**95.65**
9	Credit	49.79	71.93	61.10	72.76	62.38	73.94	67.14	80.72	**68.08**	**82.00**

**TABLE 3.** The comparison of accuracy and F1 score of several feature processing methods for Ensemble as the classifier in comparison to IFL features. The numbers in the parenthesis in front of PCA shows the threshold of variance. The numbers in the parenthesis in front of RICA and SF shows the ratio of the number of learned features related to the number of original features that are added to the original features. The numbers in the parenthesis in front of Relief-F, Fisher, Lasso, UDFS, FSV, DGUFS shows the ratio of the number of selected features to the number of original features.

DATA SETS	Cryotherapy		Heart		Segment		Glass		Magic	
Feature processing	F1	Accuracy	F1	Accuracy	F1	Accuracy	F1	Accuracy	F1	Accuracy
PCA (99%)	57.78	57.78	60.37	60.74	35.64	34.67	26.95	35.05	77.86	80.49
PCA (99.9%)	58.88	58.89	67.50	67.78	39.08	37.20	29.25	39.72	79.02	81.41
RICA (0.25)	83.28	83.33	75.13	75.19	96.40	96.47	60.00	62.62	66.16	66.91
RICA (0.5)	81.05	81.11	77.77	78.15	90.58	90.67	57.48	64.02	52.12	52.49
SF (0.25)	84.32	84.44	78.32	78.52	96.74	96.80	55.27	58.88	78.67	80.36
SF (0.5)	85.47	85.56	76.21	76.30	95.33	95.47	45.55	49.07	69.38	70.52
Relief-F (0.9)	94.43	94.44	77.42	77.78	97.56	97.60	71.34	75.70	85.88	87.43
Fisher (0.9)	94.43	94.44	76.87	77.41	97.17	97.20	70.93	75.23	84.96	86.65
Lasso (0.9)	93.30	93.33	76.35	76.67	97.56	97.60	70.71	74.77	85.90	87.49
UDFS (0.9)	92.17	92.22	78.13	78.52	97.29	97.33	71.17	74.77	85.59	87.20
FSV (0.9)	92.17	92.22	77.82	78.15	97.10	97.13	70.93	75.23	84.66	86.45
DGUFS (0.9)	94.43	94.44	76.87	77.41	97.56	97.60	70.93	75.23	84.96	86.65
IFL	**96.65**	**96.67**	**81.82**	**82.22**	**97.97**	**98.00**	**76.52**	**79.44**	**86.07**	**87.61**
DATA SETS	Diabetes		Ionosphere		Spam		Credit			
Feature processing	F1	Accuracy	F1	Accuracy	F1	Accuracy	F1	Accuracy		
PCA (99%)	65.54	69.40	80.95	82.62	64.02	65.46	49.17	73.67		
PCA (99.9%)	68.42	72.53	82.20	83.76	56.58	56.92	49.08	73.45		
RICA (0.25)	69.24	71.74	91.30	92.02	82.99	83.66	51.27	55.99		
RICA (0.5)	64.62	66.93	89.32	90.31	82.99	83.66	44.34	44.34		
SF (0.25)	69.93	72.92	77.26	77.78	93.86	94.18	60.44	70.42		
SF (0.5)	70.90	73.70	70.22	70.37	89.10	89.52	54.30	59.47		
Relief-F (0.9)	69.92	72.79	92.10	92.88	94.14	94.41	67.63	81.09		
Fisher (0.9)	68.66	71.74	92.43	93.16	94.11	94.37	67.61	81.06		
Lasso (0.9)	69.58	72.53	92.40	93.16	94.69	94.94	67.45	80.88		
UDFS (0.9)	69.98	72.79	91.80	92.59	94.41	94.68	67.61	81.06		
FSV (0.9)	68.66	71.74	92.76	93.45	94.18	94.44	67.32	80.88		
DGUFS (0.9)	69.80	72.66	92.43	93.16	94.23	94.50	66.89	80.77		
IFL	**73.31**	**76.56**	**94.99**	**95.44**	**95.44**	**95.65**	**68.08**	**82.00**		

**TABLE 4.** Comparison of the performance of the proposed IFL method with the reported results in [35] in terms of accuracy for two data sets of Segment and Diabetes. The reported results of other methods were tuned to provide their best performance.

Data sets	Segment	Diabetes
Linear ELM [36]	86.61	74.59
Deep Belief Networks [37]	95.51	78.05
Stacked Auto-Encoder [14]	95.68	77.83
DrELM	95.30	77.63
*DrELM* ^*r*^	95.79	**78.22**
IFL	**98.00**	76.56

**TABLE 5.** The description of parameters used in the proposed IFL method.

Data sets	k-fold	*r*	Feature set
Cryotherapy	15	5	1,2
Heart	10	5	1
Segment	10	5	1
Glass	15	5	2
Magic	15	15	1, 2
Diabetes	15	7	1
Credit	10	15	1
Ionosphere	15	7	1,2
Spam	5	5	2

**TABLE 6.** The comparison of elapsed time in seconds of the classifier for primary features and the time for calculation of IFL features and the classifier for IFL. It should be noted the reported times are measured based on a serial calculation for IFL. The IFL features of instances can be calculated in parallel since the features are calculated per instance and the big differences appear in data sets in which the number of instances is more than thousand instances.

#	Data sets	Primary Features	IFL
1	Cryotherapy	0.496	1.65
2	Heart	0.550	3.2
3	Segment	0.921	66.8
4	Glass	0.481	8.44
5	Magic	2.98	822
6	Diabetes	0.59	10.02
7	Ionosphere	0.66	7.06
8	Spam	1.706	101.4
9	Credit	4.57	1151
